# Protein-Protein interaction network analyses of human WNT proteins involved in neural development

**DOI:** 10.6026/97320630015307

**Published:** 2019-04-30

**Authors:** Sween Dahiya, Vandana Saini, Pawan Kumar, Ajit Kumar

**Affiliations:** 1 Toxicology and Computational Biology Group, Centre for Bioinformatics, M. D. University, Rohtak, Haryana 124001 India

**Keywords:** Frizzled, STRING, Cytoscape, PPI, Cell signaling, beta-catenin

## Abstract

WNT proteins are involved from initial of neural tissue specification to the end of cell fate determination and organ development. The present work was carried out to understand the involvement of different WNT isoforms (WNT3a, WNT5a and WNT7b) in neural development. A total of 718, 546 and 1004 PPIs for WNT3a, WNT5a and WNT7b respectively, were predicted by STRING database with confidence score more than 0.400. A network carrying all the selected PPIs of targeted proteins was constructed by using Cytoscape by assigning source node, target node and combined score as edge attribute. A total 2268 interactions of WNT3a, WNT5a and WNT7b were predicted to be involved in multiple signaling pathways and developmental processes. 43 of 2268 PPIs were refined after analyzing role of targeted proteins specifically in brain and neural development. WNT3a, WNT5a and WNT7a were predicted to be interacting with 18, 17 and 11 proteins, respectively, with average node degree score of 1.89, 2.12 and 1.82 respectively. The CytoHubba algorithm identified WNT3a, WNT5a, and WNT7b as hub proteins in neural development ranked on the basis of EPC (Edge Percolated Component) score of 9.352, 9.258 and 8.387.

## Background

Humans are multicellular organisms developed from a single fertilized egg which passes through complex embryonic processes such as morphogenesis, neurogenesis, and organogenesis. All the developmental processes are performed by multiple genes involved in different signaling pathways. From the beginning of specification of neural tissue, neural tube development and to the end of cell fate determination and development of organs WNT-Frizzled signaling pathways are involved in each stage of the development. There are 19 WNT isoforms identified in humans 1. Individual WNT ligands and their receptors illustrate astoundingly varying functions during development by changing expression behavior during different signaling pathways 2. Likewise, WNT-β-catenin pathway is involved in primary body axis formation in most of the organisms 3 and also proved to be important for cortical and hippocampal patterning, development of dorsal thalamus and thalamocortical projections 4. WNT/PCP signaling plays key role in neural tube development and neural tube closure. WNT5a and WNT7b are found to be involved in central nervous system development 5. WNT3a is needed for dorsal characterization during the formation of neural plate and required for the formation of diencephalic organizer 5, 6 and has been observed to increase the expression of WNT8c in roastral forebrain cells with FGF8 fortification 7. β-catenin overexpression in LRP6 mutants showed the need of WNT/β-catenin signaling in neurogenesis of midbrain dopaminergic neurons 8,9. WNT/β-catenin signaling cascade has been observed at 8.5 embyonic day of mouse in telencephalon, diencephalon, mesencephalon, metencephalon, myelencephalon and spinal cord 10. Loss of function due to mutation can cause several developmental defects such as, a two missense mutations in WNT5a causes autosomal dominant robinow syndrome, a rare skeletal dysplasia syndrome 11. WNT3a deficiency causes irrevocable damage to the hematopoietic stem cell's self renewal, resulting in defects in progenitor cell differentiation 12. WNT7b can act both canonically and non-canonically by involving in convergent extension movement and increase in signaling, thus possibly leading to distorted neural development 13. Proteinprotein interactions (PPIs) are important for cell-cell communication, signaling pathways and several other biological processes and main goal behind these PPIs is to find out specific function of specific protein 14. PPI networks play a significant role in finding the molecular function of a protein, other proteins associated with the target protein and cluster of similar function genes or proteins. PPIs can be predicted by in vivo, in vitro and in silico approaches and the PPI data is increasing relevantly by different experimental techniques such as mass spectrometry, phage display and yeast two hybrid system in past decade 15. Several in silico computational algorithms have been developed to predict and correlate different type of PPI data present in various interaction databases. In current study, the computational techniques are implicated to predict the interaction network of WNT proteins involved in brain development especially in neural tube development and defects (WNT3a, WNT5a, and WNT7b) which will facilitate to develop insights into the role of WNT proteins and associated proteins during development and will pave the platform for identification of protein complexes involves in specific diseases and to predict possible drug binding targets.

## Methodology

### Literature search and data mining

Literatures were searched to enlist the proteins involved in embryonic development especially focusing on neural tube development. WNT3a, WNT5a and WNT7b were found to be involved directly or indirectly in all the neural developmental processes and hence selected for further studies. Possible interactions were identified for the selected WNT proteins using STRING v10.5 16 interaction database.

PPI network formation PPIs for WNT3a, WNT5a and WNT7b were predicted by STRING database on the basis of evidence sources such as text mining, experimental evidences, databases, co-expression, neighborhood, gene fusion and co-occurrence. Edge score was calculated on the basis of molecular action and nodes with confidence score more than 0.400. Among all predicted interaction data 17 PPIs for WNT3a, 16 for WNT5a and 11PPIs for WNT7b, were selected for further network construction and analyses, especially involved in neural tube development and brain development and sorted on the basis of evidence sources like experiments, co-expression and cooccurrence. Initially, individual networks were constructed for targeted proteins using STRING and the biological role of each node in neural tube development was specified. A network carrying all the selected PPIs of targeted proteins was constructed by using Cytoscape version 3.0.3 17 by assigning source node, target node and combined score as edge attribute.

### Topological analyses of PPI networks

Number of nodes, number of edges, PPI enrichment value, average node degree and average clustering coefficient were predicted for the PPI networks of three targeted proteins (WNT3a, WNT5a and WNT7b), using STRING analysis and each node was classified by their corresponding role in biological processes during development while the edges were directed according to the molecular function of the targeted proteins. A Cytoscape plugin algorithm - Network analyzer 18 was used to predict shortest path lengths, average clustering coefficient etc, for the directed graph as constructed earlier.

### Identification of hub proteins

Cytohubba 19 an algorithm of Cytoscape was used to calculate node scores on the basis of different criterion like MCC, DMNC, Degree, EPC, Bottle neck, EcCentricity, closeness, radiality, betweenness, stress, and clustering coefficient. Nodes were ranked on the basis of EPC and closeness node score to predict hub nodes from PPI network.

## Results and Discussion

### Construction of PPI network of WNT proteins:

Protein-protein interaction data of WNT proteins was retrieved from STRING v10.5. A total of 718, 546 and 1004 PPIs for WNT3a, WNT5a and WNT7b respectively, were predicted by STRING database on the basis of evidence sources such as text mining, experimental evidences, databases, co-expression, neighborhood, gene fusion and co-occurrence. Total 2268 interactions of WNT3a, WNT5a and WNT7b were predicted having confidence score higher than 0.400, involved in multiple signaling pathways and developmental processes like cell differentiation, regulation of catalytic activity, embryonic morphogenesis, neuronal development, tissue development, CNS formation, neuron formation etc (Supplement data 1 is available with authors). PPI's with STRING-score lower than 0.4 were not included in the study because of their low confidence score for interaction and least role in neural development processes. Among predicted interactions, PPIs having role in neuronal development were selected for each targeted protein (WNT3a, WNT5a, and WNT7b) and sorted on the basis of co-expression, co-occurrence and experimental evidences because other evidences such as neighborhood, gene fusion and databases were discarded due to zero scores for most of the interactions. Total 43 PPIs were refined after analyzing role of targeted proteins specifically in brain and neural development (Table 1).

### WNT3a, WNT5a and WNT7b Interaction Network Analysis:

**Figure 1:**
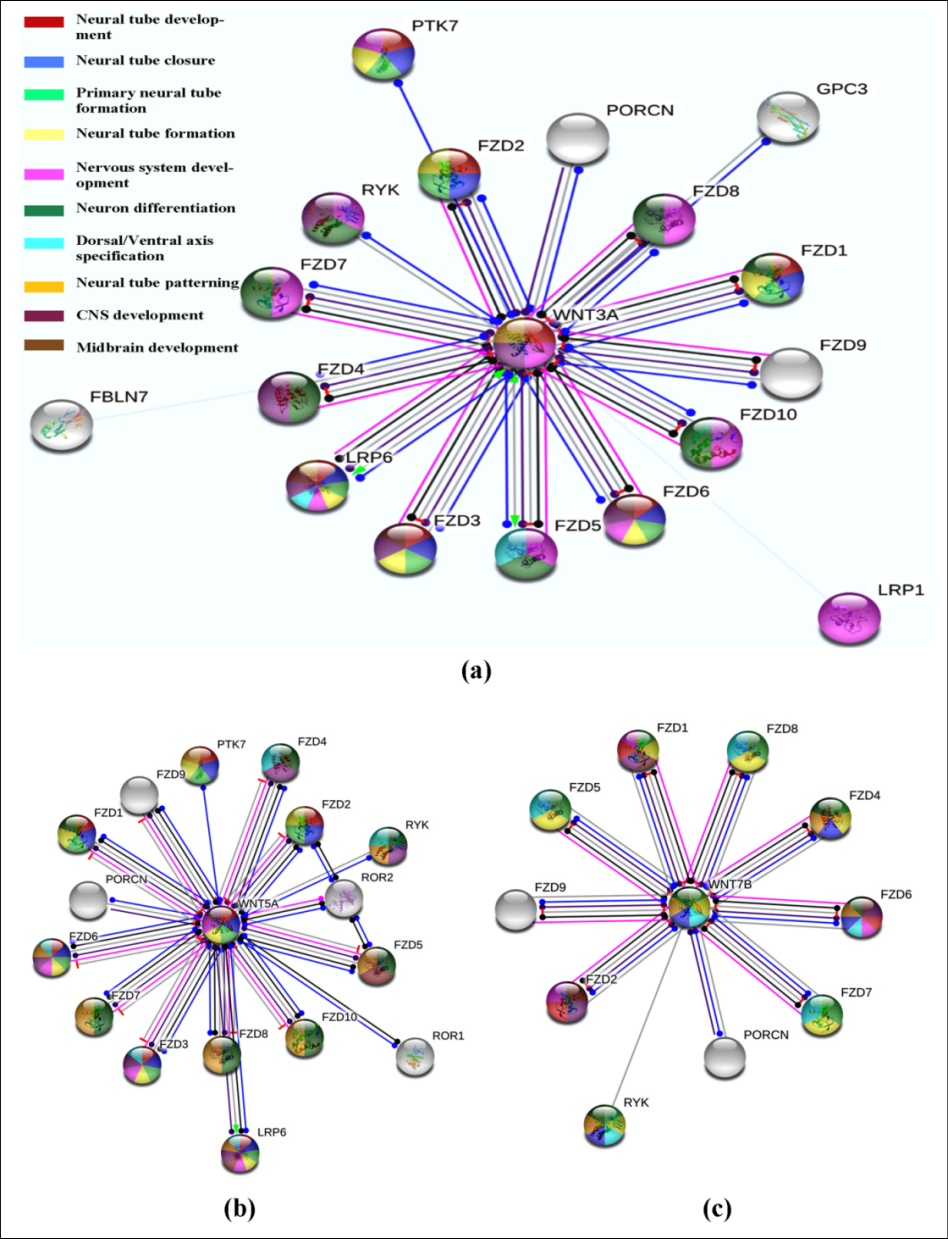
PPI network of (a) WNT3A, (b) WNT5A and (c) WNT7B as predicted by STRING (Different colors represent different neural development functions).

For all the three proteins PPI networks were constructed individually using STRING tool. WNT3a was predicted to be interacting with 18 proteins in different manner. WNT3a was illustrated as activator for LRP6 and FZD5 and inhibit the actions of FZD2, FZD1 and others as indicated by edge color predicted by STRING (Figure 1a). Nodes and edges were colored on the basis of their developmental role while the proteins involved in multiple functions were filled with multiple colors. For WNT3A, red color indicated the role in neural development and reflected that proteins FZD1, FZD2, FZD3, FZD6, PTK7 and LRP6 were involved along with WNT3a for neural tube development. Similarly, blue color nodes were depicted to have role in neural tube closure while the color representations and their corresponding functions in the study were as shown in Figure 1a. The STRING database analysis depicted that WNT3A PPI network comprised of 18 nodes connected with 17 different edges after applying relevant filters. Expected number of edges was observed to be 17 while the average node degree score was found to be 1.89 i.e., one node had at least 1.89 interacting nodes. Average local clustering coefficient was predicted to be 0.944 and PPI enrichment value was observed as 0.539. Likewise, the PPI network of WNT5a ( Figure 1b) was statistically analyzed and was inferred that there were 17 nodes in the network connected by 18 edges while each node was connected to at least 2.12 interacting nodes. The number of expected edges was found to be 16 while the average local clustering coefficient was predicted to be 0.923 with PPI enrichment value of 0.347. Similarly, the PPI network graph of WNT7b (Figure 1c) was analyzed after sorting of interacting proteins on the basis of experiments, co-expression and co-occurrence. After statistical analysis 11 nodes were found to be interacting with 10 edges having average node degree of 1.82 while the average local clustering coefficient and PPI enrichment value was predicted to be 0.909 and 0.545 respectively.

### Statistical analysis by Network Analyzer:

**Figure 2:**
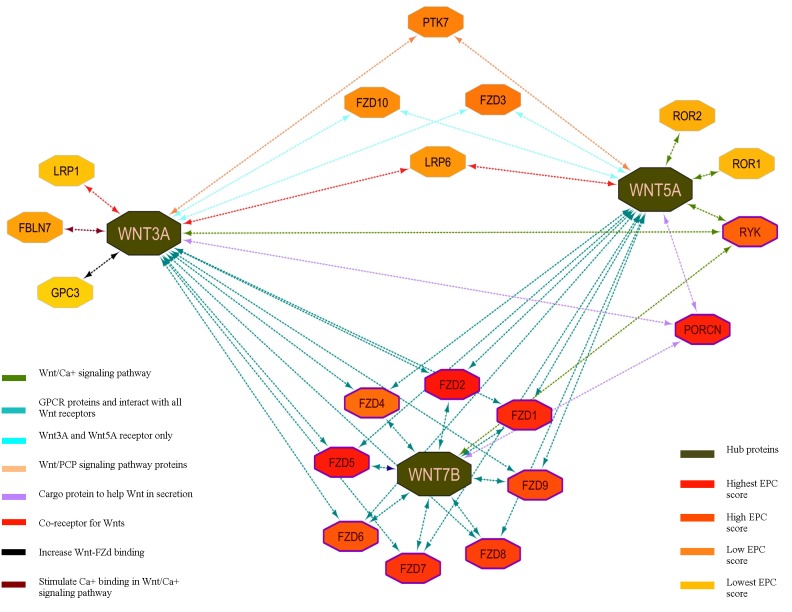
Integrative PPI network of selected 22 proteins involved in neural development functions (Nodes colored on the basis of EPC scores; Edges color represents different signaling function)

**Figure 3:**
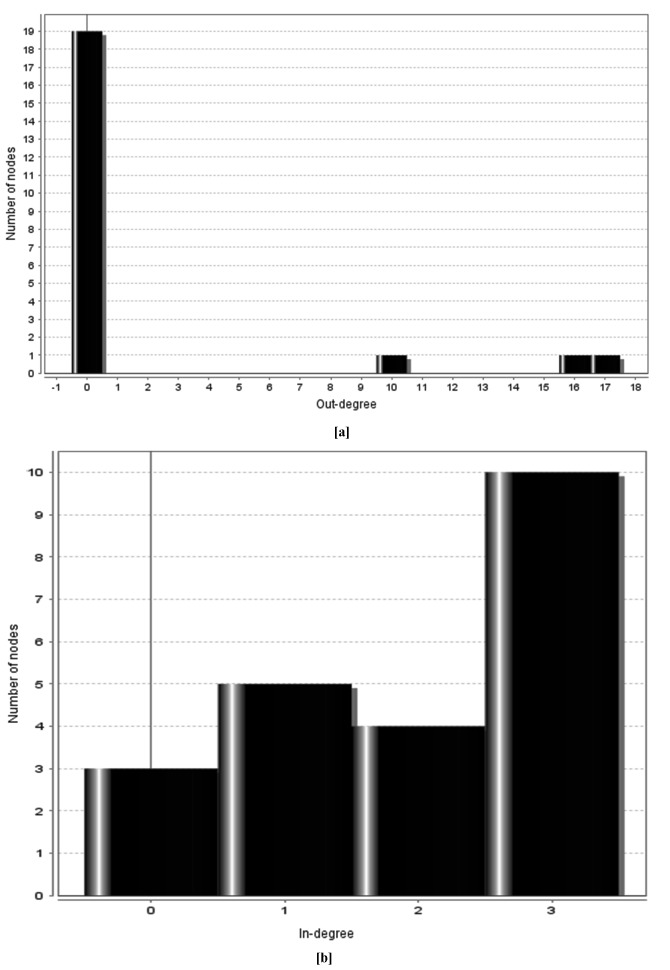
Outdegree and indegree graphs of selected 22 nodes as plotted by Network Analyzer. (a): Represents number of outgoing edges for the nodes; (b): Represents number of incoming edges for the nodes.

A common network for three targeted proteins (WNT3a, WNT5a, and WNT7b) was constructed by using software Cytoscape-3.0.3 by defining source node, target node and edge attribute. Out of 43 interactions, 22 interactions were plotted by CytoHubba algorithm of Cytoscape that identified WNT3a, WNT5a, and WNT7b as hub proteins in neural development (Figure 2; Table 2). Network graph properties such as ecCentricity, closeness of nodes, betweenness, radiality, degree etc, were calculated by CytoHubba algorithm. The targeted nodes ranking was done on the basis of EPC (Edge Percolated Component) and betweenness because each node carry different type of information and to connect nodes with each other information should be pooled and hence the betweenness was calculated to find out the relationship between the two nodes. EPC predicted the global connectivity properties of the PPI network 20 and other score like DMNC, MNC were analyzed to have insignificant and consistent values and hence neglected for the evaluations. The idea of edge percolation in a network gives a likely method for predicting major cluster structure inside a graph. Percolation method calculated the correlation score between two the nodes of a network which carried possibility to be connected with each other even after removal of some edges based on nonlocal properties of the network like short path length. Correlation calculated by EPC method has biological importance to explain the impact of one protein on another directly or indirectly in a PPI network 20. CytoHubba analyzed the PPI network of WNT proteins and ranked all nodes according to EPC score. Each node of PPI network was colored according to EPC scores of nodes with the predicted hub proteins of the network having high biological significance and evolutionarily conserved than other proteins. The WNT3a, WNT5a and WNT7b proteins with EPC scores of 9.352, 9.258 and 8.387 were predicted as hub proteins in present study (Table 3). Network Analyzer was applied on the predicted network of all WNTs for statistical analysis of the PPI network. The statistical analysis showed that a total of 22 nodes were connected with 43 edges having network radius of 1. Average number of neighbors connected was found to be 3.909. Power law was applied to the neighborhood connectivity of the nodes of graph using the formula: y=axb, where a=23.507, b=-0.80, correlation by power law was calculated to be 0.879 and R-squared value was 0.927 which clearly indicated that functional relationship between the nodes (Supplement data 2 is available with authors). Out degree and in degree graphs were also plotted for the selected 22 nodes (Figure 3a-b). In out degree distribution, total 19 nodes were observed to have out degree value of 0 indicating that the 19 nodes had no outgoing edges while the three nodes of WNT3a, WNT5a and WNT7b were observed to have 17, 16 and 10 outgoing edges, respectively. In the present investigation, the three studied nodes of WNT3a, WNT5a and WNT7b had no incoming edges while the remaining 5, 4 and 10 nodes had incoming edges of 1, 2 and 3 respectively. By in degree and out degree analyses it was examined that three of the targeted proteins were acting as hub proteins in the PPI network. The previously reported experimental PPI studies of WNTs have revealed that WNT3a interacted maximally with FZD4, FZD5, FZD7, FZD8 and transitional interaction with FZD1 and FZD2 21. Similarly, WNT5a have been reported to intermediately interact with FZD1, FZD2 and FZD4 and strongly with FZD5 and FZD8 22. Hence the our PPI network analyses had good concurrence with earlier experimental studies and revealed that the selected WNTs (WNT3a, WNT5a and WNT7b) interacted with LRP1, LRP5, LRP6, RYK and most of the FZD proteins to carry out normal cell signaling and were majorly involved in embryonic developmental activities especially in neuronal and neural plate development.

## Conclusion

The present study is a primitive but probable the first reported attempt for investigating compressively the role of WNT proteins in neural development, using in silico tools. The study revealed that WNT3a, WNT5a and WNT7b proteins are the hub proteins in neural development pathways in humans. These identified hub proteins can thus be projected at drug targets for different neural development disorders like attentiondeficit/ hyperactivity disorder (ADHD), autism, learning disabilities, intellectual disability (also known as mental retardation), conduct disorders, cerebral palsy, and impairments in vision and hearing. Our group is presently in process of further investigating these hub proteins individually for relevant drug targeting.

## Conflict of Interest

Authors declare no conflict of interest.

## Figures and Tables

**Table 1:** PPIs of selected proteins involved in brain and neural development

S. No.	Source Node	Target node	Edge attribute (Combined score)
1	WNT7B	RYK	0.678
2	WNT7B	PORCN	0.526
3	WNT7B	FZD4	0.52
4	WNT7B	FZD8	0.52
5	WNT7B	FZD5	0.52
6	WNT7B	FZD6	0.52
7	WNT7B	FZD9	0.52
8	WNT7B	FZD2	0.52
9	WNT7B	FZD1	0.52
10	WNT7B	FZD7	0.52
11	WNT3A	RYK	0.773
12	WNT3A	FZD8	0.679
13	WNT3A	FZD1	0.662
14	WNT3A	FZD2	0.642
15	WNT3A	LRP6	0.533
16	WNT3A	PORCN	0.52
17	WNT3A	FZD4	0.52
18	WNT3A	FZD5	0.52
19	WNT3A	FZD6	0.52
20	WNT3A	FZD9	0.52
21	WNT3A	FZD7	0.52
22	WNT3A	FZD3	0.52
23	WNT3A	FZD10	0.52
24	WNT3A	PTK7	0.518
25	WNT3A	GPC3	0.485
26	WNT3A	FBLN7	0.411
27	WNT3A	LRP1	0.404
28	WNT5A	FZD5	0.798
29	WNT5A	RYK	0.678
30	WNT5A	PORCN	0.667
31	WNT5A	FZD1	0.662
32	WNT5A	FZD2	0.642
33	WNT5A	ROR2	0.533
34	WNT5A	PTK7	0.532
35	WNT5A	FZD4	0.52
36	WNT5A	FZD8	0.52
37	WNT5A	FZD6	0.52
38	WNT5A	FZD9	0.52
39	WNT5A	FZD7	0.52
40	WNT5A	FZD3	0.52
41	WNT5A	FZD10	0.52
42	WNT5A	LRP6	0.479
43	WNT5A	ROR1	0.419

**Table 2:** Network graph properties as calculated by CytoHubba algorithm for proteins of selected PPIs

S. No.	Protein name	MCC	MNC	DEGREE	EPC	Bottleneck	EcCentricity	Closeness	Radiality	Betweenness	Stress
1	WNT7B	10	1	10	8.387	1	0.33333	14	3.95238	194	664
2	WNT5A	16	1	16	9.258	18	0.33333	18	3.85714	158	584
3	WNT3A	17	1	17	9.352	4	0.33333	18.66667	3.38095	3.91429	54
4	RYK	3	1	3	6.043	1	0.5	12	3.38095	3.91429	54
5	ROR2	1	1	1	3.347	1	0.25	9.91667	3.38095	3.91429	54
6	ROR1	1	1	1	3.275	1	0.25	9.91667	3.38095	3.91429	54
7	PTK7	2	1	2	5.198	1	0.33333	11.33333	3.38095	3.91429	54
8	PORCN	3	1	3	6.229	1	0.5	12	3.38095	3.91429	54
9	LRP6	2	1	2	5.024	1	0.33333	11.33333	3.38095	3.91429	54
10	LRP1	1	1	1	3.185	1	0.25	10.16667	3.38095	3.91429	54
11	GPC3	1	1	1	3.13	1	0.25	10.16667	3.38095	3.91429	54
12	FZD9	3	1	3	6.068	1	0.5	12	3.38095	3.91429	54
13	FZD8	3	1	3	6.133	1	0.5	12	3.28571	30	90
14	FZD7	3	1	3	6.14	2	0.5	12	3.28571	1.71429	24
15	FZD6	3	1	3	6.053	1	0.5	12	3.28571	1.71429	24
16	FZD5	3	1	3	6.294	1	0.5	12	3.28571	1.71429	24
17	FZD4	3	1	3	5.979	1	0.5	12	3.28571	1.71429	24
18	FZD3	2	1	2	5.282	1	0.33333	11.33333	3	0	0
19	FZD2	3	1	3	6.404	1	0.5	12	3	0	0
20	FZD10	2	1	2	5.132	1	0.33333	11.33333	3	0	0
21	FZD1	3	1	3	6.185	1	0.5	12	2.90476	0	0
22	FBLN7	1	1	1	3.376	1	0.25	10.16667	2.90476	0	0

**Table 3:** List of top 22 in network string interactions as ranked by EPC method

Rank	Protein Name	EPC Score
1	WNT3A	9.352
2	WNT5A	9.258
3	WNT7B	8.387
4	FZD2	6.404
5	FZD5	6.294
6	PORCN	6.229
7	FZD1	6.185
8	FZD7	6.14
9	FZD8	6.133
10	FZD9	6.068
11	FZD6	6.053
12	RYK	6.043
13	FZD4	5.979
14	FZD3	5.282
15	PTK7	5.198
16	FZD10	5.132
17	LRP6	5.024
18	FBLN7	3.376
19	ROR2	3.347
20	ROR1	3.275
21	LRP1	3.185
22	GPC3	3.13
